# p62 at the crossroad of the ubiquitin-proteasome system and autophagy

**DOI:** 10.18632/oncotarget.13805

**Published:** 2016-12-07

**Authors:** Victoria Cohen-Kaplan, Aaron Ciechanover, Ido Livneh

**Affiliations:** Technion Integrated Cancer Center, The Rappaport Faculty of Medicine and Research Institute, Technion-Israel Institute of Technology, Haifa, Israel

**Keywords:** proteasome, ubiquitin, autophagy, p62, protein degradation

Aberrations in the ubiquitin-proteasome system (UPS) underlie the pathogenesis of numerous diseases, many malignancies among them. Therefore, understanding its mode of regulation and the fate of its components is of broad biomedical implications. In our recent work, we show that the 26S proteasome, the catalytic arm of the UPS, which is responsible for the degradation of numerous proteins following their ubiquitination, is itself subjected to autophagy which is further stimulated under stress. The process is dependent on specific subunits polyubiquitination, and the ubiquitin chains are recognized by the shuttling protein p62, which delivers the proteasome to autophagosomes for degradation (proteaphagy) [1].

While much is known regarding the various mechanisms by which the UPS regulates different cellular processes, little is known about the fate of its own components, among them the proteasome - a large 2.5 MDa complex, or how the predator becomes a prey? Since autophagy was already shown to selectively uptake whole organelles such as mitochondria [2], we hypothesized that it can also mediate the removal of the 26S proteasome.

We show that in mammalian cells, following amino acid starvation, the 26S proteasome is recruited to autophagosomes which leads to its degradation. Importantly, proteaphagy is seen also under basal metabolic conditions, and stress stimulates the process significantly [1], which suggests that this is the main turning over mechanism for the complex. We found that amino acid starvation stimulates ubiquitination at specific sites of the 19S subunits Rpn1, Rpn2, Rpn10 and Rpn13, and showed that polyubiquitination is required for the autophagic uptake of the proteasome: silencing of the ubiquitin-activating enzyme, E1, and overexpression of non-polymerizable ubiquitin, inhibited stress-induced proteaphagy [1]. It should be noted that proteaphagy was described earlier in Arabidopsis and yeast [3,4].

An interesting novel finding relates to the role of p62 in the process. p62 is known to have a dual role in intracellular proteolysis: (i) due to its ubiquitin-associated (UBA) domain which binds a ubiquitinated cargo, and its LC3-interacting region (LIR) which recognizes the autophagic receptor LC3, it shuttles ubiquitinated targets to the autophagic machinery. (ii) another domain, Phox and Bem1p (PB1) facilitates p62 ability to bind also the proteasome, and thereby shuttle soluble ubiquitinated substrates for proteasomal degradation [5].

**Figure 1:**
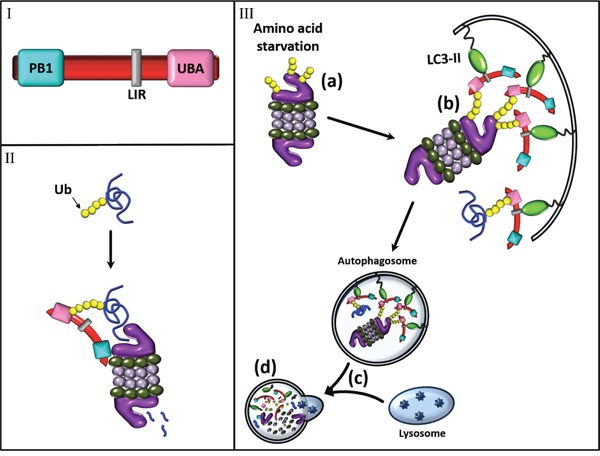
Role of p62 in shuttling ubiquitinated proteins to the proteasome and in stress-induced proteaphagy **I.** Structure of p62. p62 recognizes ubiquitinated substrates via its UBA domain and the proteasome via PB1 domain. LIR denotes the LC3 binding domain. **II.** p62 targets ubiquitinated proteins to the 26S proteasome. **III.** p62-mediated stress-induced proteaphagy. Amino acid starvation stimulates ubiquitination of specific proteasomal subunits **(a).** leading to their recognition by the p62 UBA domain, and to subsequent binding – in a similar manner to other ubiquitinated targets – to LC3 (via the LIR domain) in the evolving phagophore **(b).** Following its maturation, the autophagosome fuses with a lysosome to generate an autophagolysosme **(c).** where proteasomes and other cargoes are degraded by hydrolases **(d)**.

We found that in addition to the PB1-mediated interaction between the proteasome and p62, which leads to proteasomal degradation of ubiquitinated substrates, starvation-induced ubiquitination of the proteasome promotes its recognition by the p62 UBA domain, rendering the proteasome a substrate for autophagy (Figure 1). Furthermore, we show that the two different interactions between p62 and the proteasome are independent, as while the interaction leading to shuttling of ubiquitinated proteins requires the PB1 domain, the stress-induced association and the subsequent autophagic uptake of the proteasome, relies on the p62 UBA domain [1].

Taken together, our findings highlight p62’s pivotal role, suggesting it may act as a “decision making” switch - being able to regulate the “flux” of substrates to both proteolytic machineries, but also to promote the degradation of components of one by the other. An obvious mechanistic and intriguing question is how this occurs: how p62 “decides” which substrate to target to which pathway; are there substrates that can be targeted to both systems - and under which conditions; and how p62 discriminates between the proteasome as a predator - the complex which degrades ubiquitinated substrates, and the proteasome as a prey - a substrate for autophagy.

The UBA domain of p62 shows a preference to K63-linked polyubiquitin chains [5]. It is likely however, that the soluble ubiquitinated substrates shuttled by p62 to the proteasome are modified mostly by K48-based chains. Thus it is possible that conformational change (induced by phosphorylation for example) and selective binding to one of the two specific chain types, determines the preference of p62 to either pathway. Of note is that binding of the p62 PB1 domain to the proteasome occurs via the Rpn10 19S subunit, which we showed to be ubiquitinated under stress. This does not seem to be coincidental, as the ubiquitination may sterically hinder binding of the PB1 domain to the proteasome, thus facilitating binding of the p62 UBA domain to the ubiquitinated proteasome, directing it to the autophagic machinery.

Interestingly and independently of its roles in the UPS and autophagy, recent study has shown that p62 plays a suppressive role in the pathogenesis of liver inflammation, fibrosis, and malignant transformation [6]. That by binding and enabling heterodimerization of the Vitamin D Receptor (VDR) and the Retinoid X Receptor alpha (RXRα), a heterodimer which is important in maintaining the hepatic stellate cells (HSCs) in a quiescent state, preventing their activation.
